# Volatile organic compounds in pea plants: a comprehensive review

**DOI:** 10.3389/fpls.2025.1591829

**Published:** 2025-09-10

**Authors:** Sara Avesani, Umberto Castiello, Laura Ravazzolo, Bianca Bonato

**Affiliations:** 1 Department of General Psychology, University of Padua, Padova, Italy; 2 Department of Agronomy, Food, Natural Resources, Animal and Environment DAFNAE, University of Padua, Padova, Italy

**Keywords:** volatile organic compounds, pea plant, *Pisum sativum*, legumes, pulse crop, sampling techniques, analytical techniques, real-time detection

## Abstract

Legumes are crops of significant global importance for ensuring food security, promoting sustainable production, and enhancing cropping efficiency within diverse agroecosystems. The pulse pea (*Pisum sativum* L.) is highly valued for its protein and micronutrient content, ranking third in global cultivation after soybeans and beans, with production mainly present in temperate regions. Pea production can be afflicted by crop losses due to biotic and abiotic stress factors, leading to an increased demand for improved defense systems. To cope with environmental stresses, plants have evolved several defense mechanisms, including the production of volatile organic compounds (VOCs), which are key in plant-to-plant communication and environmental interactions. Despite the growing interest in the characterization of plant VOCs in legumes, there has been a paucity of research on the emissions and functional roles of VOCs produced by peas, either constitutively or in response to various factors such as insects, pathogens, drought, and heat. In this review, we focused on the emission patterns and potential biological functions of VOCs produced by peas in response to various stimuli. Moreover, we discuss mass spectrometry techniques currently used or with potential applications for the study of pea VOCs. The emission of specific VOCs in response to external stimuli suggests a precise involvement in pea defense mechanisms. What emerges from this essay is that further functional studies are needed to enhance and exploit the potential of VOCs for sustainable applications, such as insect repellency, pathogen inhibition, and signaling in pea plant defense.

## 1 Introduction

The global agrifood chain is facing serious and critical challenges, including reaching food security, mitigating climate change, and meeting the increasing energy demand (Stagnari et al., 2017; Horril et al., 2024). To address these challenges, central attention must be paid to the development of sustainable food production and consumption systems (Stagnari et al., 2017; Horril et al., 2024). In this context, food legumes and legume-based production systems assume great relevance because they provide multiple services aligned with sustainability principles (Singh, 2017; Stagnari et al., 2017).

Legumes play a central role in food systems as a key source of plant proteins, carbohydrates, vitamins, and minerals for both human and animal consumption, with growing importance for human health (Graham and Vance, 2003; Stagnari et al., 2017; Drewnowski and Conrad, 2024). Legumes contribute to production systems by fixing atmospheric nitrogen, making them suitable for low-input cropping (Stagnari et al., 2017; Drewnowski and Conrad, 2024). Moreover, they help to mitigate greenhouse gas emissions, having a low carbon footprint and a reduced energy demand (Stagnari et al., 2017; Drewnowski and Conrad, 2024). Additionally, at the cropping system level, they enhance agroecosystem diversity, break pest and disease cycles, and help address plant protein deficits worldwide (Stagnari et al., 2017). Given their importance, protecting pulse crops is essential for ensuring food security (Drewnowski and Conrad, 2024; Togola et al., 2024). Pulse production is affected by quantitative and qualitative crop losses due to biotic (such as insect pests, crop diseases, and parasitic weeds) and abiotic stressors (drought, heat, and low soil fertility; Togola et al., 2024). Among these, insect pests exert the most detrimental impact on pulse crop productivity worldwide, with the diversity and severity of pest attacks varying across crops and regions (Togola et al., 2024).

Plants have evolved complex strategies to counter these threats, including the emission of volatile organic compounds (VOCs) as a defensive response to environmental stressors (Baldwin, 2010; Loreto and Schnitzler, 2010; Pierik et al., 2014). Plant VOCs are small molecules characterized by low molecular weight and high vapor pressure that quickly evaporate to reach their biological targets (Dudareva et al., 2013; Widhalm et al., 2015; Adebesin et al., 2017). Volatiles are emitted from leaves, flowers, and fruits into the atmosphere and from roots into the soil, and they may be either constitutive (continuously emitted) or induced (elicited by stresses or during specific developmental stages; Loreto and D’Auria, 2022). Plant VOCs belong to a broad range of chemical classes, such as terpenoids (including hemiterpenes [C_5_], monoterpenes [C_10_], sesquiterpenes [C_15_], homoterpenes [C_11_ and C_16_], and diterpenes [C_20_]), phenylpropanoids and benzenoids, fatty acid derivatives (including green leaf volatiles [GLVs], such as C_6_- and C_9_-volatile aldehydes), and amino acid derivatives (Dudareva et al., 2006, 2013). Terpenoids constitute the largest class of secondary metabolites and derive from two common hemiterpenes: isopentenyl diphosphate and its allylic isomer, dimethylallyl diphosphate (Dudareva et al., 2013; Bergman et al., 2024). These two hemiterpenes are substrates for the prenyltransferases enzyme, which produce geranyl diphosphate (the ten-carbon precursor of all monoterpenes), geranylgeranyl diphosphate (the twenty-carbon precursor of all diterpenes), and farnesyl diphosphate (the fiveteen-carbon precursor of all sesquiterpenes) (Dudareva et al., 2013; Bergman et al., 2024). The formation of the hemiterpenes building units occurs through two independent pathways, such as the mevalonic acid and Frontiers in Plant Science 02 frontiersin.org methylerythritol phosphate (Bergman et al., 2024). Phenylpropanoids and benzenoids are the second largest class of plant VOCs (Dudareva et al., 2013), which originate from the aromatic amino acid phenylalanine, through seven reactions of the shikimate/phenylalanine biosynthetic pathway (Maeda and Dudareva, 2012). Benzenoid compounds originate from transcinnamic acid through either a CoA-dependent-*β*-oxidative pathway, a CoA-independent-non-*β*-oxidative pathway, or via a combination of both (Dudareva et al., 2006). Volatile fatty acid derivatives, such as C_6_ and C_9_ aldehydes or methyl jasmonate, arise from C_18_ unsaturated fatty acids, linoleic or linolenic, through the two branches of the lipoxygenases (LOX) pathway (Dudareva et al., 2013). Plant VOCs can also be amino acid derivatives, when synthesized from non-aromatic amino acids such as alanine, valine, leucine, isoleucine, and methionine (Dudareva et al., 2006). The huge variety of the volatiles produced represents the language that plants use to interact with their surrounding environment (Dudareva et al., 2006; Das et al., 2013; Karban et al., 2014; Rosenkranz and Schnitzler, 2016; Midzi et al., 2022; Bonato et al., 2021). The production and emission of plant VOCs are tightly regulated through vacuolar sequestration, vesicle transport, extracellular excretion, extracellular biosynthesis, and the storage of VOCs as inactive non-volatile glycoside precursors within cells (Midzi et al., 2022). In particular, the emission can be due to a mechanical disruption of storage structures that directly facilitates the release of VOCs into the atmosphere (Midzi et al., 2022), or to a spontaneous emission by which VOCs cross the plasma membrane, hydrophilic cell walls, and cuticle with a mechanism that remain poorly understood (Widhalm et al., 2015). Another topic requiring further investigation is how plants perceive and uptake VOCs released into their environment (Ninkovic et al., 2021; Kessler et al., 2023). Two primary hypotheses have been proposed. The first, termed the “passive perception hypothesis”, posits that VOCs dissolve in the cell membranes of recipient plant tissues due to their lipophilic properties (Kessler et al., 2023). The second hypothesis suggests a more active and specific perception mechanism (Kessler et al., 2023; Aratani et al., 2023). Evidence indicates that certain VOCs enter the cytosol of receiver plants and undergo metabolism to produce direct defensive compounds or phytohormones (Kessler et al., 2023).

The analysis and comprehension of VOC emissions and perception provide a promising avenue for real-time health status monitoring in plants as well as early diagnosis of pest infestations or pathogen infections, enabling timely interventions and the implementation of effective control measures to minimize losses (Jud et al., 2018; Tholl et al., 2021; Kheam et al., 2024). Currently, there is limited knowledge about the volatile emissions of pulse crops, particularly from pea plants. The pulse pea (*Pisum sativum* L.) is of great interest because it presents the highest protein food value among pulses, according to the nutrient-rich food index (Fulgoni et al., 2009; Drewnowski and Conrad, 2024). Moreover, it is a significant source of soluble and insoluble fibers, complex carbohydrates, vitamin B, folate, minerals, saturated fat, and cholesterol, essential in the human diet (Dahl et al., 2012; Kumari and Deka, 2021). Pea ranks third among the world’s most cultivated legumes (after soybeans and beans), with primary production in temperate regions (Smýkal et al., 2012; FAOSTAT, 2024). Accurate identification of pea VOCs requires reliable and sensitive analytical methods. In this respect, robust mass spectrometry (MS) techniques are being increasingly employed for this endeavor (Makhlouf et al., 2024b).

**Figure 1:**
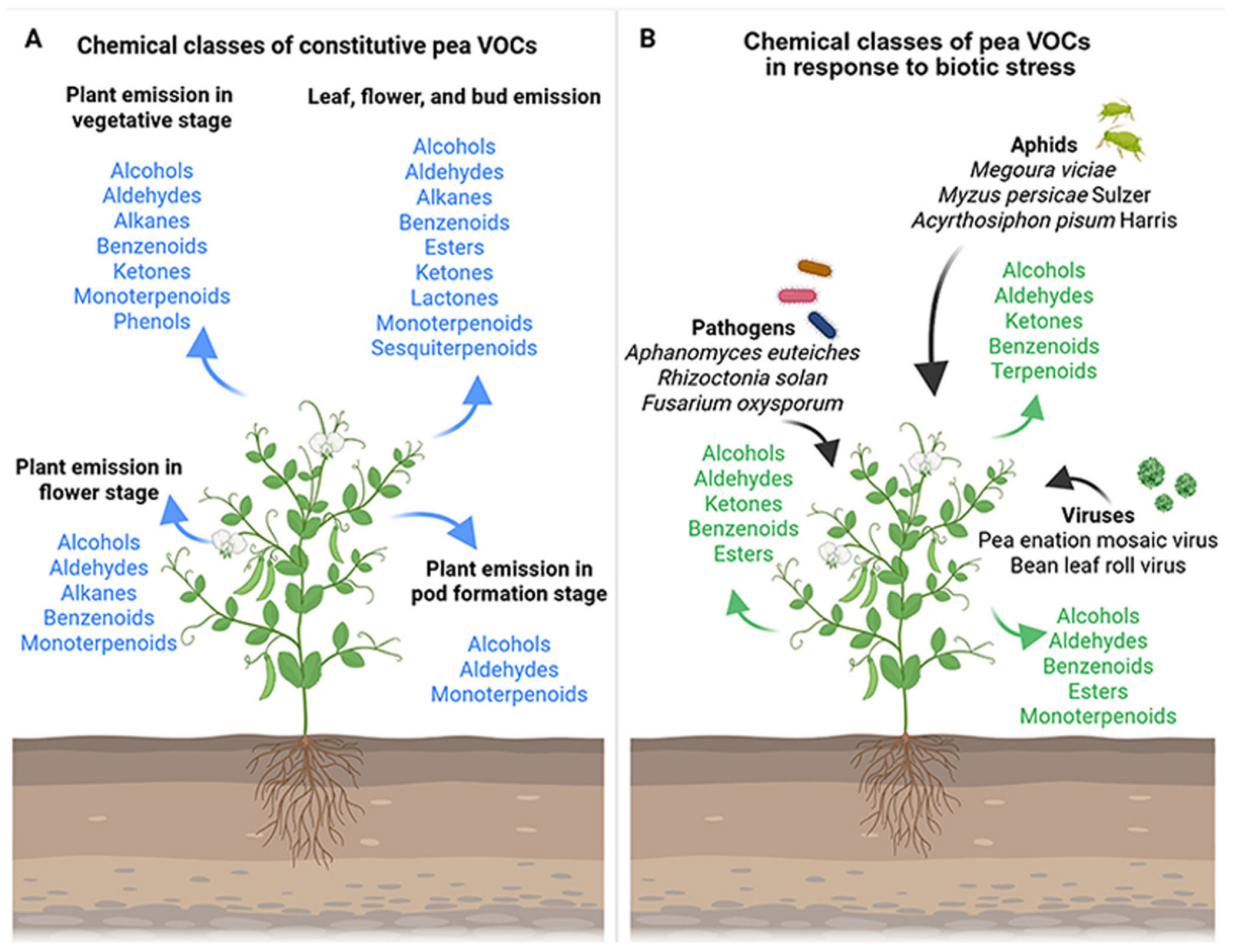
Overview of the volatile organic compound (VOC) chemical classes emission of pea plants in response to environmental stimuli. (A) Chemical class of constitutive VOCs: light blue arrows highlight the chemical classes of VOC emitted. (B) Chemical class of pea VOCs in response to biotic stress: black arrows indicate the external stimuli that affect pea VOC emission; green arrows highlight the chemical classes of VOC emitted. The figure was created with Biorender (https://www.biorender.com/).

This review is focused on the current understanding of VOC emissions in *Pisum sativum*. The first section provides a detailed discussion of pea emissions, thoroughly cataloging VOCs reported in the literature as either constitutive emissions or stress-induced responses to specific environmental stimuli (Figure 1; Tables 1, 2). In addition, this work examines VOC emissions from other pulse crops to identify common patterns and emphasize the ecological importance of plant VOC research for optimizing legume cultivation and crop selection strategies. Furthermore, the review presents an overview of advanced technologies for studying VOCs and pea metabolomics, highlighting emerging insights and offering directions for future research. Finally, it outlines why the identification and characterization of VOCs in peas hold critical significance, not only for agricultural practices but also to address broader challenges related to food security and environmental sustainability.

## 2 Volatile organic compounds in pea plants

The pea (genus: *Pisum*, subfamily: *Faboideae*, tribe: *Fabeae*) belongs to the Fabaceae family, which represents the third-largest family of flowering plants and constitutes the second most economically significant family of crop plants after the Poaceae (Graham and Vance, 2003; Smýkal et al., 2012; FAOSTAT, 2024). Among legumes, pea is consumed globally by both developed and atrisk populations. This makes it a critical economic and nutritional crop that can help increase global health and fight malnutrition (Powers and Thavarajah, 2019). Peas are naturally rich in iron and zinc and thus could address the most common micronutrient deficiencies in the world, also known as “hidden hunger” (Amarakoon et al., 2012; Lowe, 2021). Additionally, pea crop production offers several agronomic benefits, such as its role in climate change mitigation and its ability to perform symbiotic nitrogen fixation (Phillips, 1980; Smýkal et al., 2012; Stagnari et al., 2017).

Despite their potential role in precision agriculture (Brilli et al., 2019), the VOC emissions of peas remain underexplored compared to other plant species, such as grapevine (*Vitis vinifera*; Algarra Alarcon et al., 2015; Avesani et al., 2023; Chitarrini et al., 2017; Lazazzara et al., 2018, 2022; Ricciardi et al., 2021; Štambuk et al., 2023), apple (*Malus domestica*; Mehinagic et al., 2006; Espino-Dıáz et al., 2016; Wu et al., 2022; Baldi et al., 2024), and strawberry (*Fragaria × ananassa*; Neri et al., 2015; Ulrich et al., 2018; Wang et al., 2019; Li et al., 2021a; Xu et al., 2024). To fill this gap, the current work is intended to systematize VOC emissions in peas under diverse ecological conditions.

### 2.1 Constitutive volatile organic compounds in pea plants

The constitutive VOCs emission reported for *Pisum sativum* L., considering the whole plant, consist of aldehydes, benzenoids, ketones, GLVs, and monotepenoids (i.e., 2,6-dimethyl-hept-5-en-1-al, 2-hexen-1-ol, 2-propanone, 6-allyl-o-cresol, camphor, D-limonene, ethylbenzene, *n*-tridecane, *o*-cymene, *o*-xylene, *p*-xylene, α-fenchene; Giorgi et al., 2015; Figure 1A). The production and emission of volatile compounds are developmentally and compartmentally regulated in plants (Dudareva and Pichersky, 2000; Gershenzon et al., 2000; Niederbacher et al., 2015). Likewise, pea VOC content differs in emissions, considering various phenological stages and specific organs (Table 1). In particular, benzenoids, GLVs, and terpenoids emitted in the vegetative stage (e.g., 1-methylbutyl-benzene, 2,4-hexadienal, 3-carene, camphene, hexanal, limonene, myrcene, *n*-dodecane, terpinolene, α-pinene, β-pinene, [*Z*]-2-hexen-1-ol), flower stage (e.g., 1-[*S*]-verbenone, 1-methylbutyl-benzene, 2,4-hexadienal, 3-carene, camphene, hexanal, limonene, myrcene, *n*-dodecane, terpinene, terpinolene, α-pinene, β-pinene, [*Z*]-2-hexen-1-ol), and pod formation stage (e.g., 2,4-hexadienal, limonene, myrcene, *n*-dodecane, α-pinene, β-pinene, [*Z*]-2-hexen-1-ol) showed qualitative and quantitative analytical differences in *Pisum sativum* L. Var. Ambassador (Ceballos et al., 2015). Terpenes were the most abundant compounds at all phenological stages (Ceballos et al., 2015). The highest compound concentration was found in vegetative and flower stages, which coincides with Dudareva et al.’s (2004) report, in which they indicated that the emission of volatiles increases in young leaves and flowers prepared to be pollinated (Ceballos et al., 2015). Indeed, large amounts of compounds were detected at the flower stage, and in particular, terpinene and 1-*S*-verbenone were found only at this stage (Ceballos et al., 2015). Pea pod formation emitted small quantities of (*Z*)-2-hexen-1-ol, 2,4-hexadienal, α-pinene, β-pinene, myrcene, and limonene, but not *n*-dodecane, which was the dominant compound at this stage (Ceballos et al., 2015). The quantitative differences in volatile compounds emitted by flowers may be particularly important to attract pollinators (Custódio et al., 2006).

Thöming et al. (2014) specified several constitutive VOCs on the basis of the organs in *Pisum sativum* L. cv. AVOLA. As outlined in Table 1, specific VOCs were differentially found in leaves, buds, flowers, and pods. The most abundant compounds reported in all tissues were the two GLVs (*Z*)-3-hexenyl acetate and (*Z*)-3-hexen-1-ol (Thöming et al., 2014). In particular, detached pea flowers and flowering pea plants emitted large amounts of (*E*)-*β*-ocimene and (*Z*)-*β*-ocimene (Thöming et al., 2014). Additionally, pea plants undergoing leaf development emitted high levels of (*E*)-3-hexen-1-ol and methyl salicylate, and plants with pods released large amounts of (*Z*)-3-hexenal and (*E*)-3-hexen-1-ol (Thöming et al., 2014). The volatiles specific to pea flowers and buds elicited antennal responses in mated *Cydia nigricana* females, and they can be considered candidate compounds potentially involved in pea moth host location (Thöming et al., 2014). These findings suggest the crucial involvement of ubiquitous plant volatiles in pea moth host locations by the pea moth and in broader plant–environment interactions (Thöming et al., 2014).

### 2.2 Volatile organic compounds in response to environmental stressors in pea plant

VOCs in plants have been primarily studied considering the interactions between plants and herbivory insects or pathogens (Pierik et al., 2014; Heil, 2014; Sharifi et al., 2018, 2021; Hammerbacher et al., 2019; Moreira and Abdala-Roberts, 2019; Zhou and Jander, 2022). In this regard, research on plant-emitted volatiles has predominantly been focused on biotic stresses, while other environmental conditions remain comparatively underexplored (Sharifi et al., 2018, 2021; Hammerbacher et al., 2019; Ninkovic et al., 2021). The specific literature on *Pisum sativum* L. reflects this trend, with several studies reporting VOC emission in response to attacks by aphids, viruses, or pathogens (Figure 1B; Table 2; Mai et al., 2014; Wu et al., 2014; Giorgi et al., 2015; Bera et al., 2020; Song et al., 2021; Oliete et al., 2022; Marzougui et al., 2022).

The VOCs emitted by peas as a defensive response to aphid attacks seem to be aphid-specific (Mai et al., 2014; Wu et al., 2014; Giorgi et al., 2015; Bera et al., 2020; Song et al., 2021). An example is given by the emissions of *Pisum sativum* L. in response to the attack of aphids *Megoura viciae* ([*2E*]-3-pentyl-2,4-pentadien-1-ol, 2,3-squalene-epoxy, 4-isopropyl-5-methylhexa-2,4-dien-1-ol, cyclohexanol,1-methyl-4-[1-methylethenyl]-,[*Z*]-, limonene, squalene; Song et al., 2021; Figure 1B; Table 1) and *Myzus persicae Sulzer* (1-hexanol, 2,6-dimethyl-hept-5-en-1-al, 2-hexen-1-ol, 2-propanone, 6-allyl-o-cresol, aromadendrene, camphor, D-limonene, ethylbenzene, *m*-xylene, *n*-tridecane, *o*-cymene, *o*-xylene, *p*-cymene, *p*-xylene, pinocarvone, tetradecanal, α-copaene, α-fenchene, α-patchoulene, α-phellandrene, α-pinene, β-caryophyllene, [*Z*]-*β*-terpineol; Giorgi et al., 2015; Figure 1B; Table 2). Likewise, the accumulation of some potentially volatile hormones and signals (i.e., ethylene, jasmonic acid/methyl jasmonate, nitric oxide, salicylic acid) was influenced in intensity and duration in *Pisum sativum* L. cv. Cysterski in response to aphid *Acyrthosiphon pisum Harris* (Mai et al., 2014; Figure 1B; Table 2), supporting the specificity of the pea response to different aphid attacks.

Moreover, the attack of viruses could enhance the emission of specific VOCs (Wu et al., 2014; Bera et al., 2020). For instance, attacks of pea enation mosaic virus (PEMV) or bean leaf roll virus (BLRV) and *Acyrthosiphon pisum Harris* could elicit in *Pisum sativum* L. cv. Aragon (Figure 1B; Table 2) the emission of GLVs and terpenoids, such as 1-hexanol, β-ocimene, β-pinene, acetic acid hexyl ester, (*E*)-3-hexen-1-ol, (*E*)-3-hexenyl butyrate, nonanal, (*Z*)-3-hexen-1-ol, and (*Z*)-3-hexenyl acetate (Wu et al., 2014).

Additionally, in *Pisum sativum* cv. Banner and *Pisum sativum* cv. Dark Skinned Perfection, jasmonic acid and salicylic acid in response to PEMV and *Acyrthosiphon pisum Harris* (Bera et al., 2020; Figure 1B; Table 2) have been found. Despite the similarity of the emitted compounds, the composition of the blend, the number of individual volatiles, and the emission time are probably cue specific (Sharifi et al., 2018).

Regarding *Pisum sativum* responses to pathogens, recent experiments have been focused on pea plants (*Pisum sativum* L. var. Ariel, var. Hampton, var. Crécerelle [G1706325] and Firenza [N14139]) (Marzougui et al., 2022; Oliete et al., 2022). To better explain, *Pisum sativum* L. var. Ariel and var. Hampton have been found to emit (*E*)-2-hexenal, (*Z*)-3-hexen-1-ol, hexanal, nonanal, and (*Z*)-3-hexenyl acetate in response to a common water mold, *Aphanomyces euteiches* Drechs (Figure 1B; Table 2; Marzougui et al., 2022). Moreover, 1-hexanol, 1-octanol, 1-octen-3-ol, 1-pentanol, 2-octanone, 2-pentyl-furan, 3,5-octadien-2-one, 3-octanone, benzaldehyde, and hexanal have been found to be emitted in *Pisum Sativum* L. Crècerelle in response to *Aphanomyces euteiches, Rhizoctonia solani*, and *Fusarium oxysporum* (Oliete et al., 2022; Figure 1B; Table 2). The differences in VOC emission in response to the same pathogen *Aphanomyces euteiches*, depending on the variety of pea, indicate the specificity of the signal and the ways the compound is combined to convey the same message within the same species differently (Oliete et al., 2022).

Concerning abiotic factors, no evidence of VOC emissions in *Pisum sativum* L. has been reported so far. However, some studies have documented the differential emission of inorganic compounds, such as ethylene, methane, and nitric oxide in response to abiotic stresses (Abdulmajeed and Qaderi, 2019; Leshem and Haramaty, 1996; Kolbert et al., 2005). For instance, Abdulmajeed and Qaderi (2019) described methane (CH_4_) emission patterns in response to light radiation, water deficit, and high temperature in *Pisum sativum* L. var. Sundance. Leshem and Haramaty (1996) described emissions of nitric oxide and ethylene in severed and wilted plants of *Pisum sativum* L. cv. P. F. 70A. Additionally, Kolbert et al. (2005) observed nitric oxide emissions in *Pisum sativum* under osmotic stress induced by polyethylene glycol (PEG) in a nutrient solution. Further studies are needed to deepen our understanding of pea emissions in response to environmental stressors and to distinguish between species-specific and general responses. To address this, the following section will compare the responses of *Pisum sativum* to environmental stresses with those of pulse crops in general.

### 2.3 Volatile organic compounds in pea and pulse crop plants in response to environmental stressors

Volatile organic compounds emitted by herbivore-infested plants can mediate direct and indirect defense mechanisms, deterring herbivore oviposition or attracting herbivore enemies (Kessler and Baldwin, 2001; Dudareva et al., 2006; Heil, 2014; Mostafa et al., 2022; Zhou and Jander, 2022; Wu et al., 2024). In pea plants, VOC emissions following herbivore attacks primarily consist of monoterpenes, sesquiterpenes, benzenoids, and GLVs (Mai et al., 2014; Wu et al., 2014; Giorgi et al., 2015; Bera et al., 2020; Song et al., 2021; Figure 1B; Table 2). However, the functional role of VOCs in pea defense remains to be fully elucidated. Some direct repellent effects against the aphid *Megoura viciae* were shown by selected monoterpenes (such as [-]-α-pinene, [-]-*β*-pinene, and [+]-limonene) potentially produced by *Pisum sativum* L (Ceballos et al., 2015; Wu et al., 2014; Giorgi et al., 2015; Song et al., 2021). No studies have yet reported the activation of indirect defense mechanisms in peas caused by VOCs. However, an accumulation of jasmonic acid and salicylic acid was found in *Pisum sativum* L. in response to aphid attacks by *Acyrthosiphon pisum*, suggesting the induction of defense signaling in infested plants (Bera et al., 2020; Mai et al., 2014). In pulses, similar VOC-mediated defenses have been observed (Hardie et al., 1994). For example, the monoterpene (-)-(*1R*,5*S*)-myrtenal from the broad bean (*Vicia faba*) directly deterred *Aphis fabae* (black bean aphid) from selecting host plants (Hardie et al., 1994). Likewise, the benzenoid methyl salicylate, derived from salicylic acid, repelled *Aphis fabae* from broad bean (Hardie et al., 1994). Notably, methyl salicylate is one of the most important defense VOCs (Gong et al., 2023; Mahmood et al., 2024), capable of both direct insect repulsion and indirect defense activation (Gong et al., 2023; Mahmood et al., 2024). It is primarily released by plants in response to insect infestation and can trigger systemic acquired resistance (SAR) in nearby plants, leading to increased insect repellence or enhanced attraction of natural enemies, thereby reducing the insects’ survival fitness (Gong et al., 2023; Mahmood et al., 2024). This kind of indirect defense mechanism, promoted by tri-trophic plant–herbivore–carnivore interactions (Abdala-Roberts et al., 2019), has been documented in several legumes, including bean, fava bean, lima bean, cowpea, pea bush, and pigeon pea (Romeis et al., 1997; Takabayashi and Dicke, 1996; Agbessenou et al., 2018; Colazza et al., 2004). However, it remains still unexplored for pea. Indirect defense strategies promoted by herbivore-infested plants’ VOCs also include the expression of defense-related genes and the emission of volatiles in healthy leaves on the same plant or neighboring unattacked plants, increasing their attractiveness to carnivores and decreasing their susceptibility to the damaging herbivores (Arimura et al., 2002, 2004). For instance, in lima bean, monoterpenoids ([*E*]-*β*-ocimene), sesquiterpenoids ([*E*]-nerolidol), and homoterpenes (4,8-dimethyl-1,3,7-nonatriene, 4,8,12 -trimethyl-1,3,7,11-tridecatetraene) emissions induced up-regulation of defense-related genes (such as pathogenesis-related proteins, lipoxygenase, phenylalanine ammonialyase, farnesyl pyrophosphate synthase, ocimene synthase, and terpene synthase 2) after *Tetranychus urticae* or *Spodoptera littoralis* Boisduval infestation, suggesting that airborne signals mediated the plant–plant interactions (Arimura et al., 2000; Boggia et al., 2015). Interestingly, despite plants’ ability to defend themselves against their enemies through VOC emissions, the pea aphid (*Acyrthosiphon pisum*) can actively prevent the release of some terpenoids (i.e., β-caryophyllene, [*E*]-*β*-ocimene, and [*E,E*]-4,8,12-trimethyl-1,3,7,11-tridecatetraene) that would otherwise attract its parasitoid during feeding (Schwartzberg et al., 2011). This highlights the complexity of plant–pest interaction and underscores the need for further research to improve crop defense strategies through a deeper understanding of plant communication and chemical signaling.

It should be noted that VOCs are not always beneficial to damaged plants, and they can prove to be a double-edged sword in ecological interactions. While they often contribute to plant defense, in some cases, they can attract herbivores, leading to increased attacks on the plant (Ge et al., 2019; Wang et al., 2020; Mobarak et al., 2022; Mitra et al., 2021). For instance, the pea aphid *Acyrthosiphon pisum* Harris was attracted to virus-inoculated pea plants, which exhibited significantly higher ratios of GLVs to monoterpenes compared to non-inoculated plants (Wu et al., 2014). Interestingly, it was demonstrated that after the PEMV infection, the herbivore *Acrythosiphon pisum* induced several antipathogen plant defense signals (Basu et al., 2021). Still, these defenses were inhibited by *Sitona lineatus* feeding, suggesting how diverse communities of biotic antagonists alter plants’ defense traits through complex pathways that depend on the identity of attackers (Basu et al., 2021). However, *Acrythosiphon pisum* exposed to the monoterpene *E*-*β*-farnesene reduced its PEMV acquisition and inoculation in plants, suggesting that volatile signals may indirectly decrease the spread of plant pathogens by altering vector behavior (Lee et al., 2021). Other examples of attraction are given by the herbivore-induced GLVs and benzenoids (such as benzyl alcohol, thymol, 1-hexanol, 1,3-diethylbenzene, 2-hexenal, 2-octanol, [*Z*]-3-hexenyl-acetate, and [*Z*]-3-hexenol) for the leaf miner *Liriomyza huidobrensis*, the herbivore *Callosobruchus chinensis*, the moth *Spilosoma obliqua*, and the aphid *Aphis craccivora* Koch on bean plants, mung bean, and grass pea (Ge et al., 2019; Wang et al., 2020; Mobarak et al., 2022; Mitra et al., 2021). Although insect-attracting VOCs may not seem relevant to pulse crop defense, investigating their attraction mechanisms could be useful to develop new pest control strategies, such as the attraction of herbivores to traps (Ge et al., 2019; Wang et al., 2020; Mobarak et al., 2022; Mitra et al., 2021) or the reduction of pathogen transmission ensured by the suppression of vector populations (Lee et al., 2025).

Similar to what has been reported for VOCs emitted by herbivore-infested plants, the volatiles released in response to plant phytopathogens exhibit diverse antimicrobial properties, either by directly inhibiting microbial growth or by inducing systemic resistance (Quintana-Rodriguez et al., 2015). Pea plants infected with pathogens primarily emitted aldehydes, benzenoids, GLVs, and ketones (Marzougui et al., 2022; Oliete et al., 2022). In particular, some GLVs (such as 1-hexanol, [*E*]-2-hexenal, and [*Z*]-3-hexen-1-ol) emitted by *Pisum sativum* in response to *Aphanomyces euteiches, Rhizoctonia solani*, and *Fusarium oxysporum* displayed inhibitory activities against *Fusarium graminearum* and *Fusarium avenaceum* in chickpea plants (*Cicer arietinum*; Cruz et al., 2012). Likewise, the chickpea GLVs (i.e., 1-hexanol, 1-penten-3-ol, [*E*]-2-hexenal, [*E*]-2-hexen-1-ol, [*Z*]-3-hexen-1-ol) directly inhibited the pathogen development, showing great efficacy in *Fusarium* head blight control (Cruz et al., 2012). Similarly, some VOCs from *Phaseolus vulgaris*, such as limonene, linalool, nonanal, methyl salicylate, and methyl jasmonate, directly inhibited conidia development of the pathogen *Colletotrichum lindemuthianum*, the causal agent of anthracnose disease (Quintana-Rodriguez et al., 2015). Moreover, VOCs released by infected resistant bean plants conferred anthracnose resistance to a susceptible cultivar after being exposed for over 6 hours to volatile compounds collected from the headspace (HS) of resistant plants (Quintana-Rodriguez et al., 2015). This VOC exposure primed resistance marker genes in susceptible plants, elevating their expression levels to those observed in the resistant cultivar following pathogen inoculation (Quintana-Rodriguez et al., 2015). Thus, VOCs play a crucial role in enhancing resistance to legume pathogens by not only strengthening the defenses of the emitting plant but also influencing the resistance traits of neighboring receiver plants through induced and associational resistance (Cruz et al., 2012; Quintana-Rodriguez et al., 2015). Harnessing these mechanisms could provide new avenues for sustainable disease management and crop protection.

As regards the response to abiotic stress, the role of VOC remains poorly studied in legumes (Caparrotta et al., 2018; Salerno et al., 2017). A comparison of VOC emissions of pea plants with other legumes is currently not possible due to the lack of data on pea plants. Two studies have reported changes in VOC emission from *Vicia faba* plants in response to water and salt stress, activating multitrophic defense systems and eliciting a priming effect in neighboring plants (Caparrotta et al., 2018; Salerno et al., 2017). In addition, Tian et al. (2019) examined the priming effect of the GLV (*Z*)-3-hexeny-1-yl acetate applications in enhancing salinity stress tolerance in peanut (*Arachis hypogaea* L.) seedlings, protecting peanuts against drought stress (Tian et al., 2019). Interestingly, Vaishnav et al. (2016) demonstrated that NO improves the establishment of plant–bacteria interaction under conditions of salinity stress in soybean (Vaishnav et al., 2016). The study revealed that two VOCs (i.e., 4-nitroguaiacol and quinoline), released by *Pseudomonas simiae* bacteria and received by soybean plants, were found to enhance salt tolerance mechanism and to promote seed germination under salinity stress, highlighting the importance of plant growth promoting rhizobacteria (PGPR) in mediating plant interactions with their environment (Vaishnav et al., 2016). These findings suggest new strategies for testing attractive technologies and applications for more sustainable agriculture. However, further studies are required to clarify VOCs’ role in abiotic stress adaptation and to explore new strategies for improving crop protection.

The following section will delve into the methodologies that enable the analysis of VOCs, examining how these advanced techniques could be leveraged in future research to uncover new insights into the pea and pulse crop volatilome and how to choose the most appropriate to reach desired goals.

## 3 Analytical techniques for studying VOCs in pea plants: from sampling to analysis

The available technologies for VOC analysis provide the profile of volatile blends emitted by a plant, which could serve as an effective indicator of a plant’s health status (Tholl et al., 2006; Dudareva et al., 2006; Heil, 2014; Pierik et al., 2014; Jud et al., 2018; Tholl et al., 2021; Makhlouf et al., 2024). For this reason, the identification of VOC stress markers and their monitoring is emerging as a crucial phase in modern agricultural research and plants’ protection strategies (Tholl et al., 2006, 2021; Dudareva et al., 2006; Heil, 2014; Pierik et al., 2014; Zhang and Li, 2010; Niederbacher et al., 2015; Cagliero et al., 2021; Gan et al., 2023; Liu et al., 2023; Makhlouf et al., 2024). Special instrumentation and methodology are necessary to capture and analyze VOCs with sufficient resolution and sensitivity (Tholl et al., 2006, 2021; Materić et al., 2015; Zhang and Li, 2010). The most prevalent techniques employed for studying pea plants’ volatiles involve solvent extraction and HS VOC collection for sampling as well as gas chromatography (GC) coupled with MS (GC-MS) or flame ionization detection (GC-FID; Bera et al., 2020; Ceballos et al., 2015; Giorgi et al., 2015; Mai et al., 2014; Marzougui et al., 2022; Oliete et al., 2022; Song et al., 2021; Thöming et al., 2014; Wu et al., 2014; Tables 3, 4).

### 3.1 Sampling methods for the study of VOCs in pea plants

The sampling methods employed in the investigation of plant VOCs frequently require their collection from specific plant parts or organs (Tholl et al., 2006). This is done to distinguish the volatiles of reproductive and vegetative tissues, to ascertain stress-induced VOC emissions as local or systemic responses, or to correlate VOC emissions with tissue-specific enzyme activities (Tholl et al., 2006). VOCs are sampled either from detached plant parts or, preferably, *in situ* from enclosed plant organs to avoid additional emission of VOCs due to wounding effects (Tholl et al., 2006). The conventional sample-preparation methods for evaluating plant compound emissions involve solvent extraction, supercritical fluid extraction (SFE), and/or distillation (Zhang and Li, 2010; Cagliero et al., 2021; Liu et al., 2023). In *Pisum sativum* L., solvent extraction is carried out with *n*-hexane (Song et al., 2021; Table 3) or distilled water added with sodium chloride (Marzougui et al., 2022; Table 3) on powdered samples ground in liquid nitrogen (Song et al., 2021; Marzougui et al., 2022). Plant extraction methods focus on specific metabolites of interest to enrich the sample of various plant parts separately and to avoid the extraction of unwanted ones, but they also represent destructive and time-consuming approaches (Cagliero et al., 2021; Liu et al., 2023; Lefebvre et al., 2021). Moreover, harvesting single plant parts (e.g., flowers, leaves, fruits, roots, stems) can induce stress and thus alter the VOC profile (Cagliero et al., 2021). The analysis of plant volatiles in living systems is preferable because it provides more representative volatile emissions and reliable data by minimizing the perturbation caused by external factors, allowing the *in situ* environmental collection of whole-plant emissions (Tholl et al., 2006; Cagliero et al., 2021; Liu et al., 2023; Zhu et al., 2013). The most common approach for *in vivo* volatile collection is the analysis of the gaseous phase in equilibrium with the plant, also known as HS sampling (Tholl et al., 2006; Sgorbini et al., 2014; Cagliero et al., 2021; Table 3). The HS technique is a noninvasive, transportable, and inexpensive sampling method, but it requires working with cuvettes or chambers to create an enclosure system (Tholl et al., 2006; Materić et al., 2015; Cagliero et al., 2021; Liu et al., 2023). The chambers are usually made of VOC semineutral materials such as polytetrafluoroethylene (PTFE/Teflon), stainless steel, brass, glass, and perfluoroalkoxy (PFA; Tholl et al., 2006; Materić et al., 2015), and they can be static or dynamic to allow either static (S-HS) or dynamic (D-HS) sampling, depending on the instrumentation and procedures employed (Tholl et al., 2006; Cagliero et al., 2021; Makhlouf et al., 2024b). In S-HS, a liquid or solid sample reaches equilibrium with its vapor phase, and the target analytes are transferred to the HS according to their partition coefficients (Sgorbini et al., 2014). The term “static” implies the absence of airflow in the sampling chamber, making the volatile fraction in the HS representative of the sample emissions (Tholl et al., 2006; Sgorbini et al., 2014; Huang et al., 2019). In the case of plant analyses, VOCs emitted by vegetal tissue freely diffuse from the sampled environment to a collected medium (Makhlouf et al., 2024b). These molecules are captured on an adsorbent support positioned in close proximity to the plant (Makhlouf et al., 2024b). The static sampling techniques involve the adsorption and subsequent thermal desorption of compounds from an inert fiber coated with various adsorbents of differing polarity and thickness, tailored to the type and concentration of the targeted compounds (Tholl et al., 2021). These adsorbent phases are composed of diverse polymers such as polydimethylsiloxane (PDMS), polyacrylate (PA), or polyethylene glycol, as well as porous polymers such as divinylbenzene (DVB) or carboxen (CAR; Jansen et al., 2011; Makhlouf et al., 2024b). A significant advancement in static HS sampling is the development of solid-phase microextraction (SPME), a technique that combines diverse polymers and enables rapid and straightforward collection of volatiles from different matrices, such as fruits, flowers, leaves, stems, roots, and seeds, with the detection limits reaching the parts per billion by volume (ppbv) range (Zhu et al., 2013; Huang et al., 2019; Makhlouf et al., 2024b). Static HS sampling has been applied for the study of the volatile fraction in pea plants, but also in bean plants, cowpea plants, and green gram cultivars by using charcoal filters or SPME fibers of DVB/CAR/PDMS (Giorgi et al., 2015; Oliete et al., 2022; Colazza et al., 2004; Quintana-Rodriguez et al., 2015; Mitra et al., 2021; Wu et al., 2024). For instance, for *Pisum sativum* L. VOC collection, a plant was enclosed in a polyethylene cage, into which a manual SPME holder was inserted to extract the HS (Giorgi et al., 2015). Volatile compounds were collected using a 50/30 μm DVB/CAR/PDMS fiber with an exposure of 4 hours (Giorgi et al., 2015). The main advantages of this extraction are its simplicity, versatility, and ease of automation (Cagliero et al., 2021). Its main limit is the absence of analyte enrichment or accumulation, causing low sensitivity (Cagliero et al., 2021).

In dynamic HS sampling, a controlled and inert gas flow is passed through or over the plant sample in the HS container and directed to a trapping system, where the volatiles are concentrated by adsorption in a packed cartridge (Tholl et al., 2006; Cagliero et al., 2021). Then, the trapped volatiles can be eluted from the adsorbent matrix using solvents or thermal desorption techniques for subsequent GC analysis (Tholl et al., 2006). In particular, dynamic sample systems could include “pull and push-pull systems” and “closed-loop stripping,” described thoroughly by Tholl and colleagues (2006, 2021). Usually, the airflow passes through the growth chamber, and the air exiting the chamber crosses a door equipped with SPME fiber or a volatile substance trap consisting of tubes filled with a polymeric adsorbent, such as thylvinylbenzene and divinylbenzene copolymer (Arimura et al., 2004; Schwartzberg et al., 2011; Wu et al., 2014; Thöming et al., 2014; Ceballos et al., 2015; Wang et al., 2020; Marzougui et al., 2022; Table 3). The dynamic approach was adopted for *Pisum sativum* cv. Aragon and cv. AVOLA as well as for *Pisum sativum* L. var. Ambassador, var. Ariel, and var. Hampton (Wu et al., 2014; Thöming et al., 2014; Ceballos et al., 2015; Marzougui et al., 2022; Table 3). For instance, whole plants of *Pisum sativum* cv. Aragon were incubated in a sealed glass guillotine chamber, where charcoal-filtered air was delivered into the chamber at a rate of 300 mL/min for a period of 4 hours (Wu et al., 2014). Air exited the chamber through a port fitted with a volatile collection trap consisting of borosilicate tubing packed with 10 mg of adsorbent polymer (Wu et al., 2014). VOCs were then eluted with dichloromethane by the traps (Wu et al., 2014). Likewise, pots with *Pisum sativum* L. var. Ariel and var. Hampton plants were placed individually inside a 10 L glass with a top cover containing two gas connectors and one septum port for the insertion of a polydimethylsiloxane/ divinylbenzene SPME fiber (Marzougui et al., 2022). Filtered air was circulated inside the chamber at 50 mL/min for one hour, allowing volatile molecules to be adsorbed onto the SPME fiber (Marzougui et al., 2022). Additionally, aerial parts of *Pisum sativum* L. var. Ambassador (including leaves, flowers, and pods) were enclosed in a 900 mL Pyrex glass chamber, and volatiles were absorbed on a porous polymer adsorbent fiber (Ceballos et al., 2015). The air was dried, purified, and drawn through a glass chamber (Ceballos et al., 2015). Volatiles were extracted from the fiber by elution with hexane (Ceballos et al., 2015). Likewise, leaves, buds, flowers, and pods of *Pisum sativum* cv. AVOLA were separately placed in a 2 L glass jar (Thöming et al., 2014). Charcoal-filtered air was pushed through the jars at a rate of 220 ml/min and then through an adsorbent filter rinsed with hexane and methanol (Thöming et al., 2014). Headspace collection was completed over 3 hours (Thöming et al., 2014).

A new, promising approach to investigate plant volatile emissions, though never applied to pea plants, involves direct contact between the extraction phase of the sampling device and the plant surface (Cagliero et al., 2021; Liu et al., 2023). This approach has mainly been used to determine *in vivo* emissions using the direct immersion (DI)-SPME technique and direct contact sorptive extraction (DC-SE) obtained with PDMS tapes (Kfoury et al., 2017). DI-SPME is a minimally invasive, solvent-free technique in which a fiber coated with a sorbent material is directly introduced into the plant; with DC-SE, a PDMS tape is placed on the plant with a glass coverslip, avoiding PDMS–air interactions (Boggia et al., 2015; Kfoury et al., 2017; Cagliero et al., 2021). Boggia et al. (2015) described an example of DC-SE using tape (DC-STE) for sampling volatiles emitted during plant–insect interactions of lima bean in response to the herbivory larvae of the Mediterranean climbing cutworm (*Spodoptera littoralis* Boisduval). DC-STE is a sorption sampling technique employing nonadhesive polydimethylsiloxane tapes, which are placed in direct contact with a biologically active surface (Boggia et al., 2015). DC-STE was found to be a reliable method for the topographical evaluation of plant responses to stresses (Boggia et al., 2015). If applied to pea plants, this technique is promising because it includes *in vivo* and reproducible sampling, ease of execution, and preservation of plant material for further studies.

### 3.2 Analytical methods for the study of VOCs in pea plants

After sampling, the next step in determining the volatile profile of a sample is the analysis (Tholl et al., 2006; Materić et al., 2015). The most commonly used technique for quantitative and qualitative analysis of pea plant VOCs is GC-FID or GC-MS (Giorgi et al., 2015; Wu et al., 2014; Ceballos et al., 2015; Thöming et al., 2014; Oliete et al., 2022; Marzougui et al., 2022; Song et al., 2021; Table 4). GC is the preferred method for most applications involving pea plants and other pulse crops, as it enables the separation, characterization, and quantification of individual compounds within a sample (Makhlouf et al., 2024b; Cruz et al., 2012; Quintana-Rodriguez et al., 2015; Mitra et al., 2021; Mobarak et al., 2022; Wang et al., 2020; Arimura et al., 2004; Wu et al., 2024; Boggia et al., 2015; Schwartzberg et al., 2011; Giorgi et al., 2015; Wu et al., 2014; Ceballos et al., 2015; Thöming et al., 2014; Oliete et al., 2022; Marzougui et al., 2022; Song et al., 2021). Usually, a GC instrument consists of a temperature-controlled oven (capable of rapid and reproducible temperature ramping from ambient to over 300 °C), pressure control systems, and interfaces for sample introduction and detection (Materić et al., 2015). Inside the oven, an open tubular column with a stationary-phase film separates compounds based on their physical and chemical properties (Materić et al., 2015). Samples enter the column through a heated inlet and are transported by an inert carrier gas, such as helium, which is used in all pulse crop VOC analysis (Materić et al., 2015; Makhlouf et al., 2024b). Each of the VOCs interacts differently with the stationary phase of the column and is differentially retained. Thus, various VOCs come out of the column at different times (known as retention time), and after exiting the column, they may be identified and quantified by a detector, such as via FID or MS (Materić et al., 2015). FID is commonly used for quantitative analysis because of its wide linear dynamic range, very stable response, and their sensitivity (Tholl et al., 2006). The GC-FID is a simple, low-cost method for the analysis of organic compounds, such as hydrocarbons, which are detected when burnt (Materić et al., 2015). MS detectors are the most popular type of detector for VOC analysis (Tholl et al., 2006). Compounds exiting the GC column are ionized by electron impact (EI), and the resulting charged molecules and molecule fragments are selected according to their mass-to-charge (*m/z*; Tholl et al., 2006). The GC-MS method allows the identification of compounds by comparing the fragmentation spectra of sample molecules with those recorded in reference libraries, such as that of the National Institute of Standards and Technology (NIST; Materić et al., 2015). However, the precise annotation of a compound requires a comparison of the experimental fragmentation spectrum with the fragmentation spectrum of an authentic reference standard (Materić et al., 2015; Godzien et al., 2018). GC-FID and GC–MS systems were used to quantify VOCs after a nondestructive sampling (dynamic HS sampling) from *Pisum sativum* L. var. Ariel and var. Hampton (Marzougui et al., 2022; Table 4). The two GC systems were equipped with a ZB-1MS column (with a nonpolar phase of dimethylpolysiloxane; Marzougui et al., 2022). The oven program started at 33 °C and increased to 225 °C with a nonlinear program of 53 min (Marzougui et al., 2022). However, the preferred method for studying *Pisum sativum* VOCs is GC-MS (Giorgi et al., 2015; Wu et al., 2014; Ceballos et al., 2015; Thöming et al., 2014; Oliete et al., 2022; Marzougui et al., 2022; Song et al., 2021; Table 4). Various types of chromatographic columns have been employed in compound separation, such as HP-5 (with a nonpolar stationary phase of phenyl-methylpolysiloxane; Wu et al., 2014; Song et al., 2021), Rxi-5ms (with a low polarity stationary phase of diphenyl-dimethyl-polysiloxane; Giorgi et al., 2015), Rtx-Wax (with a polar stationary phase of polyethylene glycol; Ceballos et al., 2015), and DB-Wax (with an high-polarity stationary phase of polyethylene glycol; Thöming et al., 2014; Oliete et al., 2022) columns. Similar oven programs were used, starting at a temperature between 30 and 40 °C and increasing to between 220 and 250 °C with a nonlinear program (Giorgi et al., 2015; Wu et al., 2014; Ceballos et al., 2015; Thöming et al., 2014; Oliete et al., 2022; Marzougui et al., 2022; Song et al., 2021).

Although GC-MS and GC-FID methods are highly sensitive and can separate very similar compounds, they are offline methods and cannot detect in real time (i.e., online) the VOCs produced by the plants themselves (Liu et al., 2023). To solve this disadvantage, it is possible to apply MS techniques based on soft chemical ionization, such as SIFT-MS, PTR-MS, and AIM-MS (Materić et al., 2015; Liu et al., 2023).

SIFT-MS is a soft chemical ionization technique that utilizes chemical ionization of the VOC with H_3_O^+^, NO^+^, and O_2_^+^ as precursor ions (reagent ions). To generate precursor ions, the instrument uses water and air in a microwave resonator, producing many different ions. A quadrupole mass filter enables the user to select the desired precursor ion to enter the flow tube (a metal cylinder), where helium is used as a carrier gas. The sample is introduced into the flow tube via a heated sampling capillary with a constant helium flow (Materić et al., 2015; Smith et al., 2023). Further down the flow tube, the precursor ions react with the sample VOCs and ionize them. The ionized VOCs are filtered by the quadrupole and detected by the ion detector (Materić et al., 2015; Smith et al., 2023).

PTR-MS uses a hollow-cathode discharge source combined with a source drift region to generate reagent ions (such as H_3_O^+^, NO^+^, O^+^) that can act as proton donors with VOCs. The reagent ions obtained in the hollow cathode enter a series of metal rings (electrodes) insulated from one another, also known as drift tubes (Cappellin et al., 2013; Materić et al., 2015). The gas sample is introduced close to the beginning of the drift tube, where chemical ionization of VOCs occurs. The ionized VOCs are pulled out of the drift tube by the field generated by electrodes and focused toward the detection part of the instrument (Cappellin et al., 2013; Materić et al., 2015). The ionized VOCs are separated either by a quadrupole or by a time-of-flight (TOF) mass spectrometer and counted by a detector (Cappellin et al., 2013; Materić et al., 2015).

A recent technology based on the adduct ionization mechanism (AIM) allows sampled VOCs to be ionized via chemical ionization at medium pressures (Riva et al., 2024). The Vocus AIM reactor supports the use of many reagent ions of positive (benzene cations [C_6_H_6_^+^], acetone dimer ([C_3_H_6_O]_2_H^+^), and ammonium [NH_4_^+^]) and negative (chloride [Cl^-^], bromide [Br^-^], iodide [I^-^], and nitrate [NO_3_^-^]) polarity and is largely independent of changes in sample humidity (Riva et al., 2024). Reagent gasses and sample flow enter directly into the center of the conical reactor (Riva et al., 2024). Reagent ions are generated by compact vacuum ultraviolet (VUV) ion sources arranged radially around the central axis (Riva et al., 2024). The collision between reagent ions and VOCs allows the formation of product ions (Riva et al., 2024). At the exit of the Vocus AIM reactor, product ions are guided by a radio frequency (RF) quadrupole ion guide that efficiently focuses the analyte ions into a narrow beam toward the detector (Riva et al., 2024).

Online techniques are not yet widely applied for studying VOCs in legumes. However, the first study regarding the online monitoring of pea VOCs has recently been described (Avesani et al., 2024). The emission of two varieties of *Pisum sativum* L. (*sativum sativum* and *sativum macrocarpon*) were compared during the first stages of plant growth, revealing differences in emitted VOC species (Avesani et al., 2024). Online monitoring techniques are promising for pulse crop application because they offer many advantages, including reduced sample preparation, low detection limits, high selectivity and sensitivity, VOC variation recording, and noninvasive screening (Materić et al., 2015; Brilli et al., 2019). The main drawback concerns the absence of chromatographic separation, which causes the addition of all compounds with the same molecular weight in a single signal (Materić et al., 2015).

Although GC- and MS-based techniques are the most diffuse and exhibit excellent separation performance, high sensitivity, and selectivity, their applications are predominantly conducted under laboratory conditions. This approach facilitates the identification of novel VOCs but does not allow to capture the dynamic volatile profiles that mediate ecological interactions in natural environments (Gan et al., 2023). In order to perform field analyses, portable instruments are necessary to detect already known markers associated with plant stress conditions (Gan et al., 2023). The choice of the appropriate VOC analysis method could help researchers understand complex plant traits such as stress tolerance, disease resistance, or crop yield, which are essential for developing sustainable agriculture strategies. However, to fully understand plant VOC emission, the patterns of synergistic and/or antagonistic effects of biotic and abiotic factors affecting the plant in combination need to be further investigated and explored in depth.

## 4 Conclusion and future directions

VOCs produced by *Pisum sativum* (and by pulse crops in general) in response to environmental stimuli represent specific signaling molecules and belong mainly to benzenoids, GLVs, and terpenoids. Although studies on legume VOCs have largely focused on biotic stress responses, the emission profile can be influenced in composition, intensity, and duration by both biotic and abiotic stresses, allowing plants to interact directly and specifically with other plants or organisms. The role of VOCs in mediating plant responses, enabling plants to inhibit disease development, induce resistance to pathogens or herbivores and regulate control pest populations in the field by acting as traps.

The exploitation of VOC functions and modes of action offers new tools in the development of pea and pulse crop protection strategies within an agrifood system confronted with numerous emergencies. There is an increasing demand for food security while focusing on the use of sustainable agriculture. In this context, the application of powerful analytical techniques in studying and monitoring pulse VOCs enables prevention, early detection of pest infestations or pathogen infections, and timely intervention to minimize crop losses. Future studies on pea VOCs should include the identification of specific stress-related markers, ensuring the development of real-time, field-based analysis methods. Promising applications to employ in the study of VOCs in real-time and in the field include portable GC- and MS-based devices (Lee et al., 2016, 2018; Gan et al., 2023), electronic noses (Zhang and Li, 2010; Tholl et al., 2021; Liu et al., 2023; Gan et al., 2023), and customized nanosensors, such as electrical, gravimetric, optical, or wearable sensors (Gan et al., 2023). For instance, optical Raman scattering nanosensors were used to create a sensor plant able to detect multiple VOCs on-field (Choi et al., 2025). The study suggested that interfacing nanosensors with plants offers an innovative tool for monitoring field VOC (Choi et al., 2025). Moreover, wearable sensors were applied to plant leaves for real-time fingerprinting of VOCs, allowing noninvasive and early diagnosis of plant diseases (Li et al., 2021b). Interestingly, a PTR-TOF-MS has been employed for ambient measurements of VOC in the forest, gaining insights into the atmospheric oxidation of terpenes (Li et al., 2020).

Further functional studies are required to elucidate pea and pulse crop VOCs mechanisms of action in responses to biotic and abiotic factors. In addition, technical challenges must be addressed to enable effective field monitoring. However, tools and knowledge are increasingly becoming available to solve these shortcomings and facilitate VOC monitoring, paving the way to improved pulse crop defenses and more resilient agricultural systems.

## Figures and Tables

**Table 1:** Volatile organic compounds constitutively produced by pea plants.

Condition	Pea plant genotype	Annotated VOCs	Reference
Potted living plants	*Pisum sativum* L.	2,6-dimethyl-hept-5-en-1-al, 2-hexen-1-ol, 2-propanone, 6-allyl-o-cresol, camphor, D-limonene, ethylbenzene, *n*-tridecane, *o*-cymene, *o*-xylene, *p*-xylene, α-fenchene	Giorgi et al., 2015
Vegetative stage	*Pisum sativum* L. Var. Ambassador	(*Z*)-2-hexen-1-ol, 1-methylbutyl-benzene, 2,4-hexadienal, 3-carene, camphene, hexanal, limonene, myrcene, *n*-dodecane, terpinolene, α-pinene, *β*-pinene	Ceballos et al., 2015
Flower stage	*Pisum sativum* L. Var. Ambassador	(*Z*)-2-hexen-1-ol, 1-(*S*)-verbenone, 1-methylbutyl-benzene, 2,4-hexadienal, 3-carene, camphene, hexanal, limonene, myrcene, *n*-dodecane, terpinene, terpinolene, α-pinene, β-pinene	Ceballos et al., 2015
Pod formation stage	*Pisum sativum* L. Var. Ambassador	(*Z*)-2-hexen-1-ol, 2,4-hexadienal, limonene, myrcene, *n*-dodecane, α-pinene, β-pinene	Ceballos et al., 2015
Leaf emission	*Pisum sativum* cv. AVOLA	(*E*)- β-ocimene, (*E*)-2-hexen-1-ol, (*E*)-2-hexenal, (*E*)-3-hexen-1-ol, (*E*)-hexenyl acetate, (*Z*)-β-ocimene, (*Z*)-3-hexen-1-ol, (*Z*)-3-hexenal, (*Z*)-3-hexenyl acetate, 2-ethylhexan-1-ol, 3-hexanone, 6-methyl-5-hepten-2-one, benzaldehyde, decanal, hexan-1-ol, hexan-3-ol, methyl salicylate, nonanal, octanal, undecane, β-caryophyllene, γ-caprolactone	Thöming et al., 2014
Bud emission	*Pisum sativum* cv. AVOLA	(*E*)-β-ocimene, (*E*)-2-hexen-1-ol, (*E*)-2-hexenal, (*E*)-3-hexen-1-ol, (*E*)-hexenyl acetate, (*Z*)-β-ocimene, (*Z*)-3-hexen-1-ol, (*Z*)-3-hexenal, (*Z*)-3-hexenyl acetate, 2-ethylhexan-1-ol, 3-hexanone, 6-methyl-5-hepten-2-one, benzaldehyde, decanal, hexan-1-ol, hexan-3-ol, hexanal, hexyl acetate, methyl salicylate, nonanal, octanal, toluene, undecane, α-pinene, β-caryophyllene, γ-caprolactone	Thöming et al., 2014
Flower emission	*Pisum sativum* cv. AVOLA	(*E*)-β-ocimene, (*E*)-2-hexen-1-ol, (*E*)-2-hexenal, (*E*)-3-hexen-1-ol, (*E*)-hexenyl acetate, (*Z*)-β-ocimene, (*Z*)-3-hexen-1-ol, (*Z*)-3-hexenal, (*Z*)-3-hexenyl acetate, 2-ethylhexan-1-ol, 3-hexanone, 6-methyl-5-hepten-2-one, benzaldehyde, decanal, hexan-1-ol, hexan-3-ol, hexanal, hexyl acetate, methyl salicylate, nonanal, octanal, undecane, α-pinene, β-caryophyllene, γ-caprolactone	Thöming et al., 2014

**Table 2:** Volatile organic compounds produced by pea plants in response to biotic stresses.

Condition	Pea plant genotype	Annotated VOCs	Reference
Pathogens *Aphanomyces euteiches, Rhizoctonia solani, Fusarium oxysporum*	*Pisum sativum* L. Crécerelle (G1706325) and Firenza (N14139)	1-hexanol, 1-octanol, 1-octen-3-ol, 1-pentanol, 2-octanone, 2-pentyl-furan, 3,5-octadien-2-one, 3-octanone, benzaldehyde, hexanal	Oliete et al., 2022
Pathogens *Aphanomyces euteiches* Drechser	*Pisum sativum* L. var. Ariel and var. Hampton	(E)-2-hexenal, (Z)-3-hexen-1-ol, (Z)-3-hexenyl acetate, hexanal, nonanal	Marzougui et al., 2022
Aphid *Megoura viciae*	*Pisum sativum* L.	(2*E*)-3-pentyl-2,4-pentadien-1-ol, 2,3-squalene-epoxy, 4-isopropyl-5-methylhexa-2,4-dien-1-ol, cyclohexanol,1-methyl-4-(1-methylethenyl)-,(*Z*)-, limonene, squalene	Song et al., 2021
Aphid *Myzus persica* Sulzer	*Pisum sativum* L.	1-hexanol, 2,6-dimethyl-hept-5-en-1-al, 2-hexen-1-ol, 2-propanone, 6-allyl-o-cresol, aromadendrene, camphor, (Z)-β-terpineol, D-limonene, ethylbenzene, m-xylene, *n-* tridecane, *o*-cymene, *o*-xylene, *p*-cymene, *p*-xylene, pinocarvone, tetradecanal, α-copaene, α-fenchene, α-patchoulene, α-phellandrene, α-pinene, β-caryophyllene	Giorgi et al., 2015
Aphid *Acyrthosiphon pisum* Harris	*Pisum sativum* L. cv. Cysterski	ethylene, jasmonic acid, methyl jasmonate, nitric oxide, salicylic acid	Mai et al., 2014
Virus and aphid Pea enation mosaic virus (PEMV), Beanleaf roll virus (BLRV), *Acyrthosiphon pisum* Harris	*Pisum sativum* cv. Aragon	1-hexanol, acetic acid hexyl ester, β-ocimene, β-pinene, (*Z*)-3-hexen-1-ol, (*Z*)-3-hexenyl acetate, nonanal, (*E*)-3-hexen-1-ol, (*E*)-3-hexenyl butyrate	Wu et al., 2014
Virus and aphid Pea enation mosaic virus (PEMV), *Acyrthosiphon pisum*	*Pisum sativum* cv. Banner, *Pisum sativum* cv. Dark Skinned Perfection	jasmonic acid, salicylic acid	Bera et al., 2020

**Table 3:** Summary of advantages and drawbacks of sampling methods for studying VOC in pea plant.

Samplig methods	Methodology	Advantages	Drawbacks	Studies on *Pisum sativum*
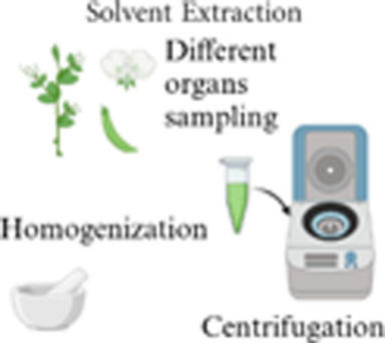	- ddH_2_O with sodium chloride extraction - methanol extraction - *n*-hexane extraction	- Distinction of specific VOCs in different vegetative organs - Sample enrichment	- Detection of additional emission of VOCs due to wounding effects - Destructive - Time-consuming approach	- *Pisum sativum* L. var. Ariel and var. Hampton (Marzougui et al., 2022) - *Pisum sativum* L (Song et al., 2021)
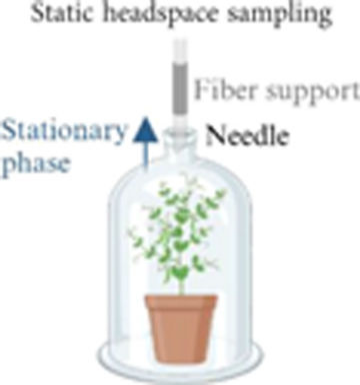	- Charcoal filters - Solid phase micro-extraction (SPME)	- *In vivo* sampling - Non-invasive method - Transportable - Inexpensive - Analysis of a representative static VOC fraction - Simple techniques	- Requires to work with cuvettes or chamber - Adsorption and subsequent thermal desorption of compounds from an inert fiber	- *Pisum sativum* L (Giorgi et al., 2015) - *Pisum sativum* L., Crécerelle and Firenza (Oliete et al., 2022)
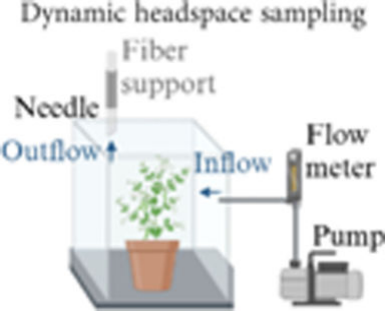	- Airflow through a growth chamber. The air exiting the chamber crossed SPME fiber or a polymeric adsorbent column	- *In vivo* sampling - Dynamic sampling with an inert gas flow - Non-invasive method - Transportable - Inexpensive	- Requires to work with cuvettes or chamber - Adsorption and subsequent thermal desorption of compounds from an inert fiber	- *Pisum sativum* cv. Aragon (Wu et al., 2014) - *Pisum sativum* var. Ambassador (Ceballos et al., 2015) - *Pisum sativum* cv. AVOLA (Thoming et al., 2014) - *Pisum sativum* var. Ariel and var. Hampton (Marzougui et al., 2022)

The figure were created with Biorender (https://www.biorender.com/).

**Table 4:** Summary of advantages and drawbacks of analytical methods for studying VOC in pea plant.

Analytical methods	Methodology	Advantages	Drawbacks	Studies on *Pisum sativum*
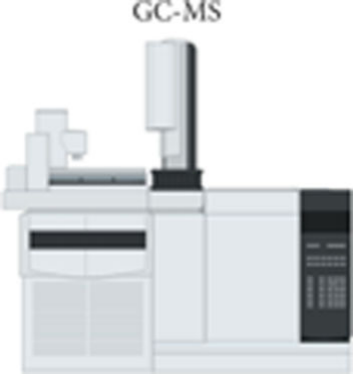	Offline	- High sensitivity and selectivity - Suitable for both qualitative and quantitative analysis	- Columns selectivity limits the total VOC estimation - Time-consuming approach - Heavy and bulky laboratory equipment - Not suitable for field applications	- *Pisum sativum* L (Giorgi et al., 2015) - *Pisum sativum* cv. Aragon (Wu et al., 2014) - *Pisum sativum* L. var. Ambassador(Ceballos et al., 2015) - *Pisum sativum* cv. AVOLA (Thöming et al., 2014) - *Pisum sativum* Crécerelle and Firenza (Oliete et al., 2022) - *Pisum sativum* L. var. Ariel and var. Hampton (Marzougui et al., 2022) - *Pisum sativum* L (Song et al., 2021)
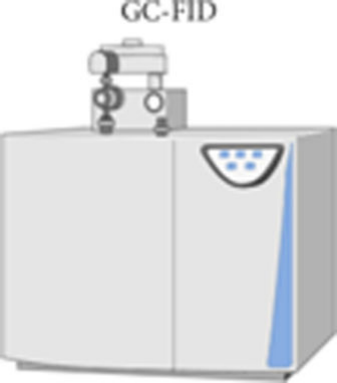	Offline	- High sensitivity and selectivity - Suitable for both qualitative and quantitative analysis	- Columns selectivity limits the total VOC estimation - Time-consuming approach - Heavy and bulky laboratory equipment - Not suitable for field applications - VOCs identification only by standard mix	- *Pisum sativum* L. var. Ariel and var. Hampton (Marzougui et al., 2022)

The figure were created with Biorender (https://www.biorender.com/).
